# How to Handle CT-Guided Abscess Drainages in Microbiological Analyses? Sterile Vials vs. Blood Culture Bottles for Transport and Processing

**DOI:** 10.3390/microorganisms9071510

**Published:** 2021-07-14

**Authors:** Romy Skusa, Christopher Skusa, Moritz Wohlfarth, Andreas Hahn, Hagen Frickmann, Marc-André Weber, Andreas Podbielski, Philipp Warnke

**Affiliations:** 1 Institute of Medical Microbiology, Virology and Hygiene, University Medicine Rostock, 18057 Rostock, Germany; hahn.andreas@me.com (A.H.); frickmann@bnitm.de (H.F.); andreas.podbielski@med.uni-rostock.de (A.P.); philipp.warnke@med.uni-rostock.de (P.W.); 2 Institute of Diagnostic and Interventional Radiology, Pediatric Radiology and Neuroradiology, Rostock University Medical Center, 18057 Rostock, Germany; christopher.skusa@med.uni-rostock.de (C.S.); moritz.wohlfarth@uni-rostock.de (M.W.); Marc-Andre.Weber@med.uni-rostock.de (M.-A.W.); 3 Department of Microbiology and Hospital Hygiene, Bundeswehr Hospital Hamburg, 22767 Hamburg, Germany

**Keywords:** drainage, pre-analysis, blood culture incubation, abdominal infections, CT, BACTEC, microbiology

## Abstract

The aim of this investigation was to compare microbiological analyses of 100 computed tomography-guided drainages from infectious foci (thoracic, abdominal, musculoskeletal), transported and analyzed by two widely established techniques, that are (i) sterile vials or (ii) inoculated blood culture bottles. The mean number of detected microorganisms from blood culture (aerobic/anaerobic) or conventional method (sterile vial, solid and broth media) per specimen were comparable with 1.29 and 1.41, respectively (*p* = 1.0). The conventional method showed a trend towards shorter time-to-result (median 28.62 h) in comparison to blood culture incubation (median 43.55 h) (*p* = 0.0722). Of note, detection of anaerobes (13% vs. 36%) and the number of detected microorganisms in polymicrobial infections (2.76 vs. 3.26) differed significantly with an advantage towards conventional techniques (*p* = 0.0015; *p* = 0.035), especially in abdominal aspirations. Despite substantially overlapping results from both techniques, the conventional approach includes some benefits which justify its role as standard approach.

## 1. Introduction

Computed tomography-(CT) guided percutaneous drainage has been the standard therapy for intra-abdominal and pelvic abscess [1]. Especially during the postoperative course, the role of CT-guided drainage as a minimally invasive procedure prevailed over surgical interventions. The main advantages of CT-supported drainage are its lower complication rate and shorter duration of drainage [2]. 

As a complication of gastrointestinal surgery, intra-abdominal abscesses lead to increased morbidity and mortality [3]. The primary goal of abscess treatment is the mechanical relief of the infectious process to avoid secondary complications [4]. The additive antibiotic therapy of abscesses is recommended for certain patient groups in order to avoid a worsening condition by peritonitis and sepsis [4]. Therefore, reliable microbiological analyses are supportive. In percutaneous puncture of abscesses, microbiological diagnostics often lead to a change in the antibiotic therapy regimen [5,6,7]. In turn, adequate antibiosis is an important condition for a positive outcome, especially for critically ill patients [8].

Microbiological diagnostics of CT-guided drainage specimens display a positivity rate between 63–85% [5,9,10,11]; abscesses associated with spondylodiscitis, for example, show significantly lower cultural positivity rates (26–42%) [6,7,12,13]. Besides the actual presence of microorganisms, positivity rates of microbiological diagnostics depend on pre-analytic conditions and techniques. If CT-guided biopsies and drainages are taken in case of suspicion of deep tissue infections, optimum quality of infectious disease diagnostics, including pre-analytic steps, is desired to justify the invasiveness of the procedure.

The value of blood culture bottle-based diagnostics has been well studied for periprosthetic infections and has indicated additional benefits of incubating tissue specimens in blood culture bottles in comparison with agar- and broth-based standard diagnostics. In detail, in a recent prospective study with patients suffering from prosthetic joint infections [14], blood culture incubation led to the detection of more pathogens than traditional growth on agar and in broths, and in several instances, microorganisms were detected in blood culture only. Another study on patients with this type of infection [15] suggested a sensitivity increase of about 47% due to additional use of blood culture bottles and also shorter times-to-result. More than 90% of blood culture isolations from periprosthetic tissue biopsies were shown to occur within 48 h [16], with a median incubation time of about 12 h [17]. In a recent meta-analysis, accuracy of blood culture-based diagnosis of pathogens in periprosthetic tissues was estimated with a sensitivity of 70% and specificity of 97% [18]. In a modeling-based assessment, cost-efficiency of the inclusion of blood culture bottles in the diagnostic workflow was suggested as well [19].

Currently, there is no gold standard in the pre-analysis of CT-guided drainage specimen handling. Therefore, transport and subsequent microbiological analyses mainly follow two regimes: either transport of the naïve specimens in a sterile vial and subsequent direct processing for microscopy and culture, or on-site inoculation of blood culture bottles followed by a single- to multi-step culture process in the laboratory. 

Different pre-analytical approaches might lead to different diagnostic results due to varying manual handling steps which might be prone to consecutive contaminations. Different preservation media, transport and atmospheric conditions of the specimen as well as the amount of inoculation volumes affect diagnostic sensitivities.

For example, using automated blood culture systems could increase positivity rates and therefore, this approach has been already implemented for analyses of primary sterile body fluids, such as liquor, ascites, synovial and sonication fluids [20,21,22,23,24,25]. Yet, analyses comparing blood culture-associated and conventional transport/culture techniques from non-sterile, thoracic, abdominal and musculoskeletal abscesses are scarce. 

In this study, 100 CT-guided drainage specimens from thoracic, abdominal and musculoskeletal foci were simultaneously inoculated onsite into blood culture bottles or transported in sterile vials and conventionally processed. Results from both approaches were compared in terms of microbial yield and time to reported result in order to give recommendations for best practice.

## 2. Materials and Methods

### 2.1. Study Design

This assessment was conducted as a single-center prospective study from April 2018 to October 2019 at the University Medicine, Rostock. A total of 100 CT-guided drainages from infectious foci were taken in the Institute of Diagnostic and Interventional Radiology, Pediatric Radiology and Neuroradiology, Rostock University Medical Center, Rostock, Germany by a radiologist. Briefly, the skin of the patients was disinfected to the hospital hygiene standards using Braunoderm (Braun, Melsungen, Germany) for at least 1 min before aspiration was performed with a 5F Unidwell needle (Bard, Heidelberg, Germany). Material from each patient underwent two transport and consecutive microbiological processing and analyses approaches in parallel, which are described in more detail below. 

### 2.2. Inclusion Criteria

Cases were continuously included in the above-mentioned period when deep-seated infection foci were suspected, and simultaneously, an indication for CT-assisted drainage was given. In addition, an aspiration volume of at least 5 mL was required to assure adequate specimen amounts for both processing and analyses modi. Only males and females who were at least 18 years old were included.

### 2.3. Descriptive Parameters

Descriptive parameters were assessed to further characterize the study group. These parameters comprised age, sex, sample acquisition site, referring ward, previous surgery or drainage, purulent appearance of the aspirate, application of chemotherapy or another immunosuppressive therapy during the last six months, Diabetes mellitus disease and antibiosis of at least 24 h at the time of drainage. The characterization of the study population is shown in Table 1. 

### 2.4. CT Imaging Protocol

After written informed patient consent, CT-guided aspiration or drainage of potentially infectious foci were performed on both inpatients and outpatients. Aspirations of thoracic, abdominal or musculoskeletal localization that yielded no fluid or less than 5 mL were not included. All CT studies were performed on a 64-slice CT-machine (Toshiba Aquilion 64, Toshiba, Neuss, Germany). Depending on clinical conditions, iomeprol (Imeron 400, 400 mg/mL Bracco Imaging, Konstanz, Germany) was injected at a flow rate of 2.5 mL/s. Thereafter, 40 mL saline solution was injected using the same flow rate. CT data acquisition was started in the portal-venous phase of enhancement. Image reconstruction was carried out at a slice thickness of 0.5 mm.

### 2.5. Collection and Specimens Processing

After the samples were obtained by aspiration, BACTEC^TM^ Plus Aerobic/F and BACTEC^TM^ Lytic/10 Anaerobic/F blood culture media (Becton Dickinson, Heidelberg, Germany) were immediately on-site inoculated with 1 mL each by a radiologist. Therefore, the seals of the blood cultures bottles were removed, and the rubber septum was disinfected for 15 s using Octeniderm (Schülke & Mayr GmbH, Norderstedt, Germany) followed by injection of the drainage fluid via an 18 G × 1 ½” (1.2 × 40 mm) blunt fill needle (Becton Dickinson, Heidelberg, Germany). The entire leftover sample material was passed into a sterile tube (12 mL tube, Greiner Bio-One GmbH, Frickenhausen, Germany). Both tubes and corresponding blood culture bottles were then transported within 2 h at room temperature to the DIN EN ISO 15189-accredited Institute of Medical Microbiology, Virology and Hygiene of the University Medicine Rostock, Germany, for subsequent microbiological analyses. There, bottles and tubes were immediately processed according to standard protocols as follows: 

Blood culture bottles were added with 2 mL of BD BACTEC^TM^ FOS^TM^ Culture Supplement (Becton Dickinson, Heidelberg, Germany). The Bactec bottles were stored and incubated for up to five days in the BACTEC FX system (Becton Dickinson GmbH, Heidelberg, Germany) according to the manufacturer’s recommendations. This culture approach is hereinafter referred to as “blood culture method”.

**Figure 1:**
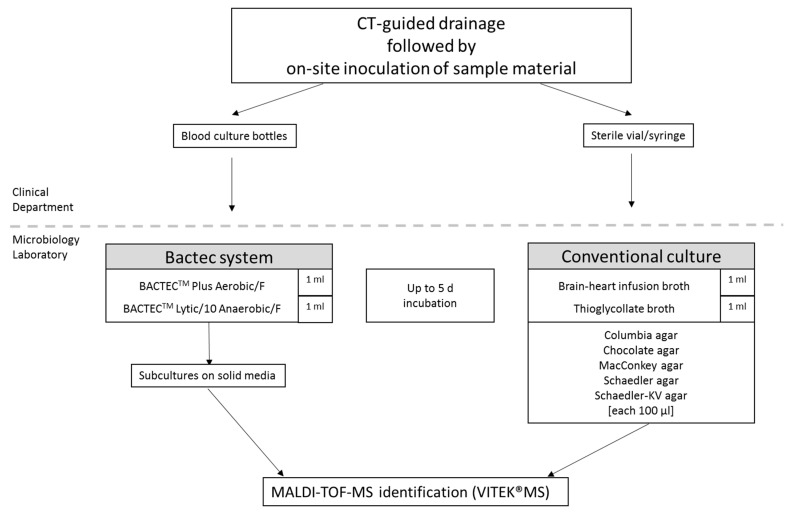
Specimens processing. Displayed are the specimen collection and processing modes. Volume information (in mL or µL) indicates the amount of specimen used for inoculation of the corresponding media.

Conventional routine culture comprised incubation on aerobic and anaerobic solid media (Columbia agar, MacConkey agar, Chocolate agar, Schaedler KV agar, Becton Dickinson GmbH, Heidelberg, Germany) as well as in liquid media (brain–heart infusion broth, thioglycollate broth, Becton Dickinson GmbH, Heidelberg, Germany), at 37 °C and 5% CO_2_/20% O_2_ (CO_2_-enriched aerobic) or 0% O_2_/10% CO_2_/10% H_2_/80% N_2_ (anaerobic) atmosphere, respectively. CO_2_-enriched aerobic and anaerobic conditions were provided by employing the KB 115 (Binder GmbH, Tuttlingen, Germany) incubator and the Anoxomat III (Advanced Instruments, Norwood, MA, USA) with appropriate jars, respectively. Disregarding the quantity of the sample in the sterile tube, standardized volumes were used for subsequent microbiological analyses. Each agar was inoculated with 100 µL specimen and each liquid culture medium with 1 mL sample to mimic the Bactec blood culture bottles inoculation amounts (Figure 1). Additionally, Gram staining from naïve specimens was performed.

Primary assessment of the agar plates was conducted after 18–24 h with subsequent daily inspection over a total incubation period of 5 days for routine culture media. This culture approach is hereinafter referred to as “conventional culture method”.

Pathogen identification was performed by MALDI-TOF-MS (Vitek MS) (bioMérieux, Marcy-l’Étoile, France) according to manufacturer’s instructions. IDs were based on determination in duplicates from two macromorphologically identical colonies each.

In case of negative results in conventional culture as well as the blood culture method, 16S ribosomal RNA (rRNA)- and 18S rRNA-gene PCR was performed from the original sample as follows: PCR amplification of the 16S rRNA gene was performed using the primers 27F (AGAGTTTGATCMTGGCTCAG) and 519R (GWATTACCGCGGCKGCTG) [26,27]. PCR amplification of the 18S rRNA gene was done using the primers S 1 (ACTGCGAATGGCTCATTAAATCAG) and CUF1 (CAAGGCCATGCGATTCG) [28,29,30]. Detected PCR products were transferred to Microsynth SeqLab (Göttingen, Germany) for single-strand sequencing according to the company’s protocols. Nucleotide sequences were compared to the NCBI database (http://www.ncbi.nlm.nih.gov/, accessed on 10 November 2019) using the Basic Local Alignment Search Tool (BLAST).

### 2.6. Outcome Parameters

Eventually, identified microflora and the diagnostic time-to-result were defined as the primary outcome parameters. In turn, time-to-result was defined as the time span between the receipt of the sample as documented in the lab IT system and the first microbe identification transmitted by matrix-assisted time-of-flight mass-spectrometry (MALDI-TOF MS).

### 2.7. Statistical Assessment

The test for proportions was used to compare blood culture and conventional culture methods in detection rate. The number of microorganisms detected by using both methods was compared with the Wilcoxon test. Data were extracted on the mean and median times-to-result for all culture positive samples and were analyzed, using the sign test. Fleiss’ kappa was used to assess the level of agreement between both culture methods. A significance level of *p* = 0.05 was defined for all calculations.

### 2.8. Ethical Clearance

The study was conducted according to the guidelines of the Declaration of Helsinki and approved by the Ethics Committee of the University Medicine Rostock (Registration number A 2018-0138). Written informed consent was obtained from all subjects involved in the study. The medical indication for the Ct-guided drainage and microbiological analyses was made by a physician independently from the study setting.

## 3. Results

A total of 100 CT-guided sample-acquisitions from 78 patients were included in the study. Of the 100 specimens, a total of 73 were culture positive by blood culture and/or conventional method. In particular, using the blood culture method, 71 specimens displayed bacterial and/or fungal growth, whereas by the conventional method 64 specimens led to this result. Culture negative samples from both methods were counterchecked by 16S- and 18S-rDNA PCR analyses as well as by microscopy, and were consistently found to be negative.

There was no statistically significant difference in the mean number of detected microorganisms per specimen, which was 1.29 (SD 1.21) with the blood culture method and 1.41 (SD 1.62) with the conventional culture method (*p* = 1.0). Moreover, when analyzing the mean numbers from subgroups of specimens ordered by sample acquisition site, no statistically significant differences were revealed. Results are summarized in Table 2. 

A polymicrobial infection (≥ 2 microorganisms) was detected in 33 samples processed by the blood culture method vs. 34 samples processed by the conventional method. The mean number of detected species in polymicrobial infections varied significantly between both approaches with 2.76 (SD 0.87) in the blood culture vs. 3.26 (SD 1.44) in the conventional setting (*p* = 0.035), respectively.

With respect to the isolated species, a total of 129 different isolates were detected by the blood culture method in comparison to 141 isolates detected by the conventional culture method. Descriptively, aerobic gram-positive cocci were most frequently detected (*n* = 65 specimens processed by blood culture method vs. 52 specimens processed by conventional method; Table 3). The most frequently isolated species was *E. coli* (*n* = 28 by blood culture method vs. 25 by conventional method). *S. aureus* and Enterococci tended to be more frequently isolated by blood culture method. For a detailed list considering the species-level of microbe identifications, please refer to Supplementary Table S1.

Overall, Fleiss’ kappa index [31] indicated substantial (0.61–0.80) agreement of overall results obtained from the two approaches, but considerable differences when focusing on individual microbial groups. While almost perfect (0.81–1.00) agreement between the two approaches was observed for aerotolerant gram-negative rod-shaped bacteria and substantial agreement for mixed bacterial cultures and yeasts, agreement was only moderate (0.41–0.60) for aerotolerant gram-positive coccus-shaped and rod-shaped bacteria, as well as for anaerobes. For the latter, there was a significantly higher detection rate in conventional culture method (Table 4).

The median time-to-result (Table 5), defined by the time span between IT-documented specimen arrival at the laboratory and the first MALDI-TOF-MS-based identification was 43.55 h for the blood culture method and 28.62 h for the conventional culture methods (*p* = 0.0722).

A total of 33 anaerobic isolates were detected with both methods. All 10 anaerobic microbes isolated by blood culture method (10; 30.3%) were also detected by the conventional method. A total of 23 additional anaerobic microbes were detected by conventional method only (23; 69.7%), resulting in a significantly higher detection rate of anaerobes (*p* < 0.0001). No anaerobic microorganisms were found in thoracic drainage material. The majority of anaerobes detected by conventional method were identified in abdominal aspirations (30; 90.9%), *p* < 0.0001 (Table 6).

## 4. Discussion

In the examined patient collective, microorganisms could be isolated from the majority of samples irrespective of the diagnostic approach. Specifically, the blood culture method detected microorganisms from 71% and the conventional method from 64% of the samples. However, this difference was not statistically significant. Focusing also on subgroups of clinical patients, anatomical drainage location or on mean number of isolates per sample, the blood culture-based and the conventional culture method did not show significant differences depicting both approaches being suitable for daily routine microbiological analyses. Of note, the comparably low number of included musculoskeletal drainage fluids makes the interpretation for this type of samples challenging. 

The present patient collective displayed a 73% overall microbe detection rate. This value was higher than in previous examinations, which employed comparable methods but dealt with samples from normally sterile body fluids, i.e., cerebrospinal, peritoneal, pleural or synovial fluid. In these studies, positivity rates ranged between 24 and 26% [20,21,32]. Studies on microbiologic diagnostics of CT-guided drainage material from abdominal abscesses led to a comparable positive detection rate ranging from 60–78%, without specifying their culture methods [10,33,34]. To our best knowledge, only one study has evaluated the blood culture method for microbiological diagnostics of brain abscesses, but not in comparison with conventional culture methods [35]. 

In the present study, employing the 16S- and 18S rDNA PCR for samples negative by culture techniques did not improve the overall detection rate for microorganisms. This method appears to be of small, if any, advantage for analyzing abscess drainage fluids. Especially in polymicrobial infections, superimposed and thus, hardly distinguishable sequences will be generated. Multiplex PCR systems, which have been successfully established for analyzing other patient materials such as cerebrospinal fluid [36], heart valve material [37,38] and respiratory specimens [39], could partly optimize microbiologic diagnostic of drainage material depending on their genomic targets. Implementing microbiome analytics in routine diagnostics might have the highest potential to overcome these drawbacks in the near future.

Considering the time span to a positive result, this study shows a promotive tendency towards the routine culture technique. For vitreous fluid, a study comparing the blood culture method with the conventional culture method did not find a difference in the time-to-microbe identification [40]. Oppositely, several studies found a significant time advantage for the blood culture method when examining cerebrospinal fluid, sonication fluid and other normally sterile body fluids [20,22,41,42].

Abdominal, thoracic and musculoskeletal abscesses differ in the spectra of causative microorganisms [43,44,45,46,47,48]. With respect to the groups of microorganisms detected by both culture methods, the conventional culture method displayed superior results in detection of anaerobes. Anaerobic bacteria constitute the majority of commensal flora, especially in the gastrointestinal and genitourinary tracts. In the case of extraintestinal infections, anaerobes could aggravate the infection process started by aerotolerant pathogens. Therefore, especially in the case of abdominal abscesses after surgical infections in these areas, the etiological significance of anaerobes must be considered, questioning the diagnostic value of identifying these bacteria. What could be the reason for the diverse sensitivity for anaerobes in both methods? First, anaerobes occur predominantly in polymicrobial foci of infection instead of causing a monomicrobial infection [49]. Second, anaerobic bacteria usually have a longer replication time than most relevant human pathogenic aerobes [50]. Thus, liquid culture systems such as the blood culture bottles could favor outcompetition of anaerobes in samples from polymicrobial infections, in contrast to solid agar media, which allow separate growth of individual colonies. An advantage of liquid sample incubation in blood culture bottles for detecting gram-positive cocci, especially enterococci, as reported in former studies [20,21] could not be confirmed in this study. Of note, slightly higher numbers of isolated *S. aureus* and Enterococci strains from the blood culture method in the present study might foster these observations.

The evaluation of isolates as etiologically relevant or potentially contaminating is complexed by the lack of a gold standard, especially if the isolate is part of the physiological flora. Therefore, it could not be completely ruled out that a better detection rate in one method might be due to a higher contamination rate. However, the latter aspect can be minimized by a rigorous hygienic regimen.

This study did not focus on economic aspects of the microbiological analyses in terms of prices of consumables or manual handling steps, because individually negotiated prices, quantity-driven retail rates and billing-specific factors such as DRG-defined gross payments would render unrepresentative data. Nevertheless, economic calculations geared to the specific local situation are important and could overrule the suggested diagnostic algorithm.

As potential limitations of this study, optimum sample transportation within 1–2 h such as in our setting, is not provided in all medical care facilities, so prolonged transport time to the laboratory could lead to an advantage towards blood culture bottle transport and incubation. Moreover, culture methods were only performed during regular working hours (7 AM to 7 PM), which may contribute to a prolonged time-to-result. Finally, direct pathogen identification from positively recorded blood culture broth as well as rapid subculture combined with MALDI-TOF identification was not yet established at the study site when the analyses were performed. Optimization of the latter two approaches could shorten the time span for species identification by the blood culture method. Oppositely, the rate of mixed species cultures in the specimen collection could impair the advantage of direct analyses.

Microbiological processing followed the standard operating procedures of the DAkkS-accredited routine laboratory in terms of identification and antimicrobial susceptibility testing, using only a limited amount of colonies. This approach has the methodological-inherent risk of missing inter- or intra-species differences by picking the “wrong” colony, which in turn could affect therapeutic choices if heteroresistance is present within a population [51]. Future techniques might circumvent this general issue in routine microbial diagnostics [52].

## 5. Conclusions

In the present study, compared to the blood culture method, the conventional agar plate method shows some advantages such as a shorter time span until species identification, and a higher detection rate for anaerobes in the microbiological diagnosis of CT-assisted drainage of abdominal abscesses. Reported benefits of the blood culture-based approach for the time-to-result [16,17] do not withstand if direct pathogen identification from positively tested blood culture materials is not performed or impaired by a polymicrobial setting. In addition, native material enables further microbiological investigations, such as Gram staining and molecular-based diagnostics, as well as biochemical tests and histopathological evaluation.

## Figures and Tables

**Table 1:** Descriptive characterization of the study group.

Parameter	
	years (±SD)
**Age**	63.18 (±15.78)
	in %
**Sex**	
Male	69
Female	31
**Sample acquisition site**	
Thoracic	21
Abdominal	69
Musculoskeletal/soft tissue	10
**Referring ward**	
General and Visceral Surgery	43
Intensive Care Unit	23
Internal Medicine	20
Urology	5
Other surgical departments	8
Other departments	1
**Previous surgery**	67
**Previous puncture sample acquisition**	13

Displayed are patient numbers characterized by epidemiological and clinical parameters. *n* = 100 specimens were included.

**Table 2:** Comparison of isolate rates between blood culture method and conventional method.

	Blood Culture Method		Conventional Method		*p*-Value
	Mean	SD	Mean	SD	
Total (*n* = 100)	1.29	1.21	1.41	1.62	1
**Sample Acquisition Site**					
thoracic (*n* = 21)	0.28	0.71	0.19	0.51	0.6250
abdominal (*n* = 69)	1.56	1.14	1.75	1.61	0.7011
musculoskeletal/soft tissue (*n* = 10)	1.50	1.50	1.60	2.11	1

Displayed are the mean isolate rates from two transport and processing approaches, in total and specifically for the corresponding subgroups of the different sample acquisition sites. SD = standard deviation. Statistical analyses assessed by sign test.

**Table 3:** Groups of microorganisms isolated by blood culture or conventional culture methods.

Microorganism	Blood Culture Method	Conventional Method
Aerobes		
Gram-negative rods	42	41
Gram-positive cocci	65	52
Gram-positive bacilli	5	4
Anaerobes	10	33
Fungi	7	11
total	129	141

Displayed are microorganisms distinguished by growth conditions (aerob/anaerob) and micromorphological appearance after Gram staining.

**Table 4:** Concordance of microbiological results between blood culture and conventional method.

	Samples Detected Positive by					
	Blood Culture Method		Conventional Culture Method			
	*n*	%	*n*	%	*p*-Value	Fleiss’ Kappa (0.95 Confidence Interval)
Total	71	100	64	100	0.2906	0.751 (0.614, 0.888)
Detection of						
>2 isolates	33	46	34	53	0.4406	0.741 (0.574, 0.908)
anaerobic bacteria	9	13	23	36	0.0015	0.447 (0.235, 0.659)
aerobic gram-negative rods	34	48	33	52	0.6698	0.968 (0.905, 1)
aerobic gram-positive cocci	51	72	41	64	0.3334	0.550 (0.330, 0.769)
gram-positive bacilli	5	7	4	6	0.8538	0.466 (0.021, 0.910)
fungi	7	10	11	17	0.2110	0.664 (0.398, 0.930)

Displayed are the number of positive patient materials detected by blood culture method or conventional culture method, in total and for subgroups. Statistical analyses of positivity rate were assessed by sign test; additionally, Fleiss’ kappa-statistic measure of agreement between both methods was performed.

**Table 5:** Time to Result.

*n* = 61	Blood Culture Method		Conventional Method		*p*-Value
	Median	IQR	Median	IQR	
	43.55 h	23.18; 47.37	28.62 h	21.67; 47.00	0.0722

Displayed are the median time-to-result of blood culture and Conventional method from positive samples. IQR = Interquartile range. Statistical analyses were assessed by sign test.

**Table 6:** Number of detected anaerobic species by conventional method depending on sample acquisition site.

Sample Acquisition Site	Anaerobic Isolates Detected by Conventional Method		*p*-Value
	Yes	No	
thoracic (*n* = 21)	0	21	*p* < 0.0001
abdominal (*n* = 69)	30	39	
musculoskeletal/soft tissue (*n* = 10)	3	7	

Displayed are the median time-to-result of blood culture and Conventional method from positive samples. IQR = Interquartile range. Statistical analyses were assessed by sign test.

## Data Availability

Data is contained within the article or Supplementary Material.

## Supplementary Materials

The following are available online at https://www.mdpi.com/article/10.3390/microorganisms9071510/s1, Table S1: Microorganisms isolated from blood culture or conventional culture method.
