# Supplementary material for: How to Handle CT-Guided Abscess Drainages in Microbiological Analyses? Sterile Vials vs. Blood Culture Bottles for Transport and Processing
Source: Microorganisms. 2021 Jul 14;9(7):1510. doi: 10.3390/microorganisms9071510 (PMC8306503; doi:10.3390/microorganisms9071510)
Supplement: Supplementary file 1 [file microorganisms-09-01510-s001.zip › microorganisms-1268712-supplementary.pdf]

microorganisms

MDPI

Supplementary Materials

Table S1 Microorganisms isolated from blood culture or conventional culture method

**Microorganism**	**Blood culture method (Positive specimens n=71)**	**Conventional method (Positive specimens n=64)**
aerobic gram-negative bacteria	42	41
Escherichia coli	27 (28)	23 (25)
Klebsiella pneumoniae	5	4
Raoultella ornithinolytica	2	2
Citrobacter koseri	1	1 (2)
Proteus mirabilis	1	2
Morganella morganii	1	1
Citrobacter freundii	1	1
Klebsiella oxytoca	1	1
Pseudomonas aeruginosa		1
Haemophilus parainfluenzae	1	1
Enterobacter cloacae	1	1
aerobic gram-positive cocci	65	52
Enterococcus faecalis	15	10
Staphylococcus aureus	12	8
Streptococcus anginosus	6	7
Staphylococcus epidermidis	5	7
Enterococcus faecium	6	3
Streptococcus constellatus	4	4
Streptococcus intermedius	3	3
Streptococcus mitis/oralis	4	2
Staphylococcus hominis	2	2
Enterococcus avium	1	1
Streptococcus agalactiae	1	
Staphylococcus lugdunensis	1	1
Staphylococcus capitis	1	1
Streptococcus salivarius	1	1
Streptococcus sanguinis	1	
Streptococcus cristatus		1
Lactococcus garvieae	1	1
Aerococcus viridans	1	
gram-positive bacilli	5	4
Lactobacillus species	2	1
Actinomyces odontolyticus	1	2
Actinomyces turicensis		1
Rothia mucilaginosa	1	

Corynebacterium tuberculostearicum	1	
Anaerobic bacteria	10	33
Bacteroides fragilis	3	9
Bacteroides thetaiotaomicron	3	4
Bacteroides ovatus		1
Prevotella bivia	1	1
Prevotella buccae		1
Prevotella oris		1
Prevotella melaninogenica		1
Cutibacterium acnes	1	1
Finegoldia magna		1
Fusobacterium mortiferum		1
Parabacteroides distasonis		1
Bacteroides caccae		1
Bacteroides xylanisolvens		1
not identified to genus/species level	1 (2)	5 (9)
Fungi	7	11
Candida albicans	3	6
Candida parapsilosis	2	2
Nakaseomyces glabrata	1	2
Candida dubliniensis	1	1
total	129	141

Displayed are patient numbers characterized by epidemiological and clinical parameters. n=100 specimens were included.
